# Lysis of tubercle bacilli in fresh and stored sputum specimens: implications for diagnosing tuberculosis in stored and paucibacillary specimens by PCR

**DOI:** 10.1186/1471-2180-7-83

**Published:** 2007-09-04

**Authors:** Divya Pathak, Soumitesh Chakravorty, Mahmud Hanif, Jaya Sivaswami Tyagi

**Affiliations:** 1 Department of Biotechnology, All India Institute of Medical Sciences, Ansari Nagar, New Delhi 110029, India; 2 New Delhi Tuberculosis Centre, New Delhi 110002, India; 3 Department of Biological Sciences, Purdue University, West Lafayette, IN, USA; 4 Ruy V. Lourenco Center for the Study of Emerging and Reemerging Pathogens, Division of Infectious Diseases, Department of Medicine, New Jersey Medical School, University of Medicine and Dentistry of New Jersey, Newark, NJ, USA

## Abstract

**Background:**

Nucleic acid amplification techniques are being used increasingly in diagnosing tuberculosis. In developing countries clinical samples are often stored for subsequent analysis since molecular tests are conducted at only a limited number of laboratories. This study was conducted to assess the speed at which mycobacteria undergo autolysis and free DNA is detected in the supernatant during low-temperature storage.

**Results:**

Eighty-seven smear positive sputa from tuberculosis patients were analysed immediately and after storage at -20°C. Timelines of 1 and 2 months were selected to assess the maximum extent of DNA loss that occurred during storage. All samples remained PCR- and smear-positive at 1 month and only 1 sample turned negative after 2 months. Bacterial lysis in the specimens was demonstrated by PCR analysis of supernatant fractions; 53% of the freshly analysed samples contained mycobacterial DNA in supernatants. PCR positivity increased significantly during storage (to 69% and 77% after 1 and 2 months of storage, respectively, P < 0.0001). Storage-associated bacterial lysis was accompanied by a decrease in smear grade status in 28 of 87 samples (P < 0.0001 after 2 months of storage) and a significant storage-associated reduction in bacterial numbers in the remaining samples.

**Conclusion:**

We conclude that (i) freshly isolated sputum contains both intact and lysed mycobacteria, (ii) lysis increased during storage and (iii) supernatant fractions routinely discarded during sample processing contain mycobacterial DNA. We propose that supernatant is a valuable sample for PCR for both fresh and stored specimens, particularly those with a low bacterial load in addition to conventional sediment.

## Background

TB is an enormous public health challenge worldwide, with an estimated global incidence of 0.136% in 2005 [1] which is likely to be aggravated by the HIV/AIDS pandemic. Therefore there is great pressure on clinical laboratories to rapidly and accurately detect and identify clinically important mycobacteria. TB is routinely diagnosed worldwide by smear microscopy and culture. In spite of its lack of sensitivity and inability to distinguish between tubercle bacilli and other mycobacteria, the former technique is widely used in TB control programmes on account of its speed, simplicity and low cost. Apart from lungs, TB also occurs in other body sites wherein the paucibacillary load often poses a significant diagnostic challenge [2,3]. Nucleic acid amplification technologies are being intensively assessed in diagnosing extrapulmonary TB owing to their speed, specificity and sensitivity [4]. However their application is limited to sophisticated laboratories in developing countries including India and samples are often stored prior to analysis. Studies that have evaluated the effect of sample shipment and storage on the performance of conventional and nucleic acids-based tests concluded that accurate diagnosis rests on minimal transport time and lowest possible storage and shipping temperatures [4-6]. Furthermore, in case of paucibacillary specimens, diagnostic methods are no different from those employed for high load or fresh samples. The inherent delay in obtaining culture results is a serious limitation of the gold standard; therefore it was proposed that when smear microscopy and DNA amplification were both positive, the diagnosis of active TB could be considered established [7]. For these various reasons PCR is being increasingly considered as an adjunct test for diagnosing TB in resource-poor settings also.

We developed a sample processing procedure, USP methodology, that is compatible with microbiology and inhibition-free PCR in both pulmonary and extrapulmonary samples [8-10]. In the present study, we assessed the effect of low temperature storage on the efficacy of *M. tuberculosis *detection in fresh and stored USP-processed sputum. The key finding was that a significant number of fresh specimens contained lysed mycobacteria and lysis increased during storage. As a consequence supernatant PCR positivity increased with sample storage. Thus supernatant is a novel and useful sample for enhancing PCR performance in clinical samples.

## Results

In a preliminary study conducted to assess the impact of sputum storage on PCR, maximum bacterial autolysis in untreated samples was noted during room temperature vs. 4°C or -20°C storage (Dudeja M, unpublished observations). The present study was designed to assess the effect of storage at -20°C of NALC-treated sputum on bacterial lysis. We chose 1 and 2 months of storage as it exceeded the likely interval between sample collection and PCR analysis. PCR and smear microscopy were employed to assess the speed of storage-associated changes in bacterial load. Bacterial lysis was confirmed by simultaneous analysis of sputum supernatants that are conventionally discarded

### Effect of sample storage on PCR

**Figure 1:**
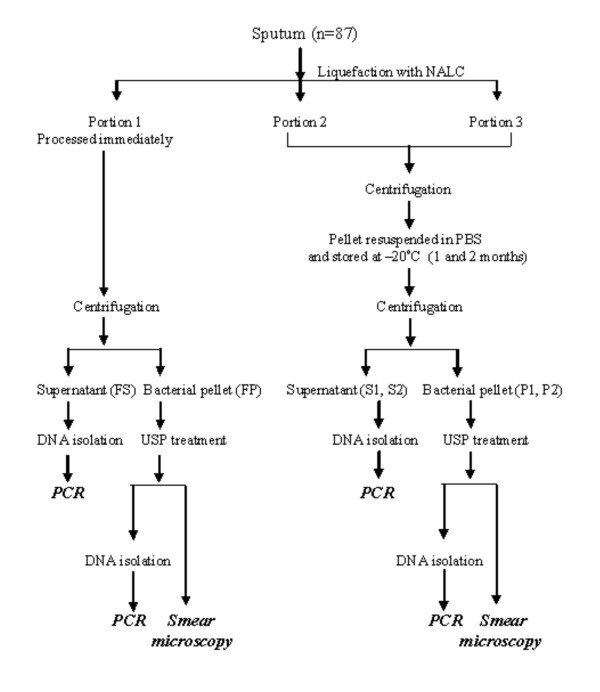
**Outline of sputum processing protocol**. FS, S1 and S2 refer to supernatant fractions from fresh, 1 month and 2 months stored samples, respectively. FP, P1 and P2 refer to bacterial pellet fractions from fresh, 1 month and 2 months stored samples, respectively.

**Figure 2:**
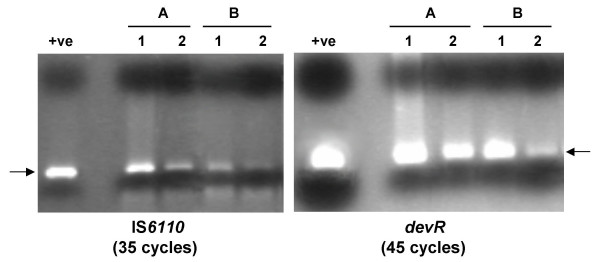
**Effect of storage on performance of IS*6110*-based PCR and *devR *PCR using pellet-derived DNA**. For both panels: +ve, purified *M. tuberculosis *DNA as positive control; lane 1, DNA isolated from fresh pellet (FP); lane 2, DNA from 2 months-stored pellet (P2). A and B represent two different samples.

All the samples were processed as outlined in Fig. 1. A total of 522 IS*6110 *PCR assays were performed on 522 sputum aliquots prepared from 87 sputum specimens (pellet and supernatant fractions of fresh and stored samples). All bacterial sediments remained PCR positive after 2 months of storage with the exception of a single sample. From these results it was inferred that bacterial lysis did not occur in smear positive stored samples to an extent that altered the outcome of PCR. The high sensitivity of PCR in stored bacterial sediments was attributed to either the initial high bacillary load (all samples were smear positive) or to the resistance of tubercle bacilli to a cycle of freeze/thaw or both. Sputum storage was associated with a decrease in DNA recovery from the sediment fractions; random analysis of a subset of samples by IS*6110 *PCR (performed for only 35 cycles) and *devR *PCR (performed for 45 cycles) indicated a reduction in DNA amplification efficiency from stored vs. freshly processed sediments (Fig. 2) which suggested the occurrence of bacterial lysis during storage.

**Figure 3:**
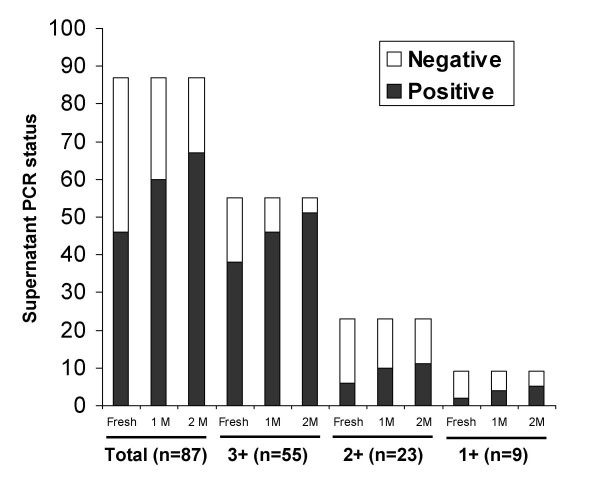
**Effect of storage on recovery of PCR-amplifiable DNA from supernatant fractions**. The number of supernatant PCR positive samples in fresh and stored samples is plotted for all samples (n = 87), initially 3+ (n = 55), 2+ (n = 23) and 1+ (n-9) samples. The increase in supernatant PCR positivity upon storage was statistically significant (P < 0.0001 among 3+ and 2+ samples and P = 0.012 in 1+ samples).

**Figure 4:**
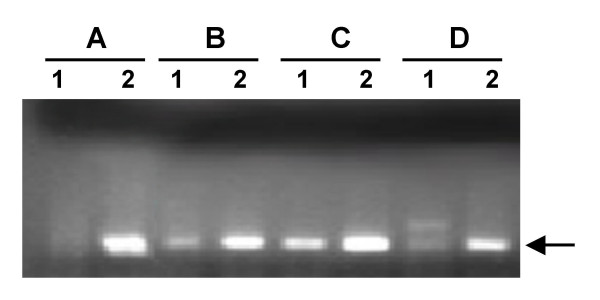
**Effect of storage on recovery of PCR-amplifiable DNA from supernatant fractions**. Lane 1, fresh supernatant (FS); lane 2, supernatant of 1 month-stored sample (S1). A, B, C and D represent four different samples.

Bacterial lysis was confirmed by *M. tuberculosis *DNA detection in the supernatant fraction that is normally discarded during processing; 46 of 87 supernatants (53%) were PCR positive among fresh aliquots. In stored samples, the number of PCR positive supernatants increased to 60 (~69%) and 67 (~77%) after 1 month and 2 months, respectively (Fig. 3) that was statistically significant (P < 0.0001). A representative result of PCR amplification from supernatants is shown in Fig. 4. We concluded that even fresh samples contained lysed bacteria and that DNA lost due to storage-associated bacterial lysis from the sediment was recovered in the supernatant fraction.

### Effect of sample storage on USP smear status

**Figure 5:**
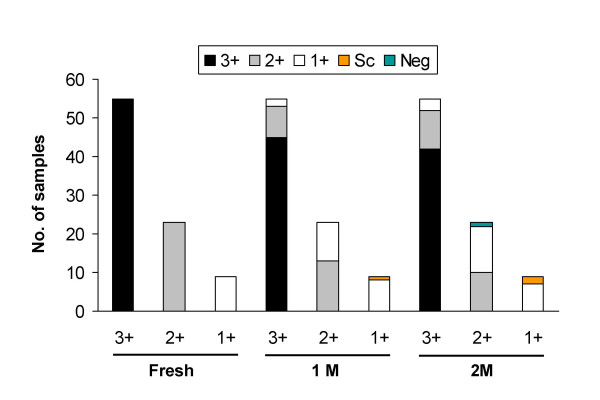
**Effect of storage on USP smear grade status of sputum samples**. The reduction in smear grade status of 3+, 2+ and 1+ samples have been shown separately. 1 M and 2 M refer to 1 month and 2 months of sputum storage at -20°C.

A total of 261 slides prepared from 87 specimens (fresh sample and after 1 month and 2 months of storage) were examined by smear microscopy for acid-fast bacilli. Each slide was independently read by at least 2 well-trained technicians in a blinded manner. The sputum were graded as 3+ (n = 55), 2+ (n = 23) and 1+ (n = 9). No sample with a scanty or negative grade status was obtained (Fig. 5). All the specimens remained smear-positive during 1 month of storage and only 1 specimen (of 2+ grade) turned smear-negative after 2 months. Thus storage and one cycle of freeze/thaw did not substantially affect USP smear positivity *per se*. However, a decrease in "smear grade" status upon storage was noted in 28 of the 87 samples which included 13 samples of 3+, 13 samples of 2+ and 2 samples of 1+ smear grades. The decrease was more evident in the case of 2+ and 1+ samples as even a small decrease in bacterial number resulted in a change in smear grade. The decrease in the number of samples of a particular grade (eg. 3+ or 2+ or 1+) upon storage was accompanied by a significant increase (P = 0.0002 and P < 0.0001) in the number of samples belonging to the lower grades (Fig. 5).

### Effect of sample storage on bacterial load

**Figure 6:**
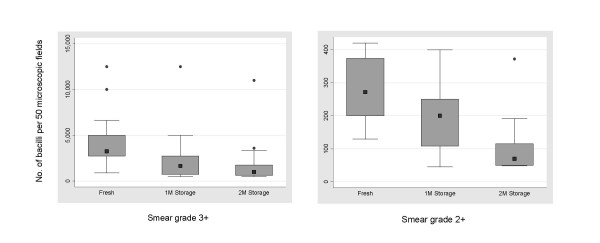
**Effect of storage on bacterial load**. Box plots of bacterial numbers per 50 microscopic fields in 3+ and 2+ slides. Boxes show median values and the interquartile ranges, the whiskers denote 2.5^th ^and 97.5^th ^percentiles and the circles indicate the outlier values. 1 M and 2 M refer to 1 month and 2 months of sputum storage at -20°C.

The remaining 59 of 87 samples that preserved their smear grade during storage were assessed for storage-associated changes in bacterial load. Thirty-seven samples showed a decrease in bacterial number. In the 3+ slides (n = 25), the median number of bacilli was 3250 in fresh samples which reduced to 1650 and 1000 after 1 month and 2 months of storage, respectively. The decrease in bacterial load from fresh to 1 month, from 1 month to 2 months of storage and from fresh to 2 months of storage were statistically significant (P < 0.001, P < 0.01 and P < 0.001, respectively). A similar trend of reducing bacterial numbers upon storage was also noted in 2+ slides (n = 9); the median number of bacilli in fresh specimens was 272 and reduced to 200 and 70 upon 1 month and 2 months of storage, respectively (Fig. 6). These changes were also statistically significant (P < 0.05 for fresh and 1 month storage, P < 0.05 for 1 month and 2 months of storage and P < 0.008 for fresh and 2 months storage). The 1+ smear grade specimens (n = 3) also showed storage associated decrease in AFB load whose statistical significance was not assessed on account of the small number of samples studied.

## Discussion

We made several noteworthy conclusions in this study that evaluated the impact of sputum storage on the speed at which bacteria underwent autolysis and released DNA in the supernatant. Firstly, our results indicate that delays of upto a month do not adversely affect PCR and smear microscopy results. Secondly, although a statistically significant decline in smear grades was observed on storage only 1 sample changed from smear-positive to -negative upon storage. The apparent negligible effect of storage on the sensitivity of smear microscopy was attributed to (i) the use of highly sensitive USP smear microscopy (detection limit ≈ 300–400 bacilli/ml of sputum [8]) and (ii) a high initial bacterial load. Thirdly, the PCR status of sediments was not altered with storage perhaps due to a high initial bacterial load, use of the multicopy IS*6110 *target assay and the addition of more template DNA in PCR (enabled by efficient inhibitor removal using USP processing, ref. [8]). The final and most significant finding was that DNA was recovered from supernatants from the majority of freshly processed samples. Additional lysis occurred during storage and was reflected by an increase in the number of PCR positive supernatants. Since the sputum was stored after NALC processing, DNA retrieved from the supernatant fraction thereafter (S1 and S2) was derived solely from bacteria lysed during storage and/or subsequent thawing. For the sample that turned smear-negative and sediment PCR-negative after storage; supernatant PCR that was initially negative turned positive after storage. Thus, the DNA lost from pellet was retrieved in the supernatant. This observation assumes significance in paucibacillary specimens that are generally subjected to a concentration step before analysis. Thus, supernatants that are conventionally discarded during sample processing would be a valuable specimen for PCR testing and offset the disadvantage associated with analyzing stored or low-load samples.

## Conclusion

We conclude that performing supernatant PCR in addition to sediment PCR and highly sensitive USP smear microscopy would increase the efficiency of TB diagnosis in case of fresh and stored paucibacillary specimens.

## Methods

### Specimens

Eighty-seven direct smear positive sputum specimens were collected from patients attending the Microbiology and DOTS centre at New Delhi Tuberculosis Centre, New Delhi before they were placed on antitubercular therapy. The left-over sputa were stored at 4°C within 5–6 hours of collection and processed within the next 6–36 hours.

### Specimen processing and storage

The sputa were processed as outlined in Fig. 1. Briefly, they were digested with 0.2 volumes of 2.5% N-acetyl L-cysteine (NALC) for 5 min and homogenized by vortexing in the presence of 5–6 sterile 3 mm glass beads for 5–10 min [11]. The liquefied sputum was distributed into three approximately equal portions. One portion (portion 1) was processed immediately by centrifugation at room temperature for 15 min at 8000 rpm (~10,000 g, GSA rotor, Sorvall RC5B Plus centrifuge, USA) to yield fresh supernatant and fresh pellet fractions (FS and FP, respectively in Fig. 1). The sediment fractions (FP) were processed by the USP method (see below) and used for smear microscopy and PCR. The remaining portions (Portion 2 and Portion 3) of NALC-treated sputum were centrifuged, the supernatants were discarded and the pellets were resuspended in sterile phosphate-buffered saline (PBS) equivalent to the original volume of the NALC homogenate and stored for 1 and 2 months at -20°C (Fig. 1).

### Universal sample processing (USP)

The sputum sediments (FP, P1 and P2) were processed by the USP method as described [9]. Briefly, to FP or P1 or P2, 1.5 to 2 volumes of USP solution (5 M guanidinium hydrochloride (GuHCl), 50 mM Tris-HCl, pH 7.5, 25 mM EDTA, 0.5% Sarcosyl, 0.15 M β-mercaptoethanol) was added. After incubation for 5–10 min the contents were centrifuged at room temperature at 8000 rpm for 15 min as above. The supernatant was discarded; the sediment was washed with water and then resuspended in 600 μl of 0.05% Tween 80. Smears were prepared on clean microscopic glass slides using 10% of the USP-processed specimen, subjected to Ziehl-Neelsen staining, examined under oil-immersion lens and graded as described [12].

### DNA isolation

DNA was isolated from the remainder of the USP-processed sediment as described [8]. Briefly 60–80 μl of resuspension solution containing 10% Chelex-100 0.3% Tween 20 and 0.03% Triton X-100 was added to the processed sediments FP, P1 and P2 (Fig. 1), mixed well and incubated at 90°C for 40 min. The extracted DNA was used for PCR.

DNA was isolated from the supernatant samples, FS, S1 and S2, (Fig. 1), using the DNA bind method [13] with minor modifications. Briefly, per volume of supernatant, 2 volumes of DNA bind solution [6 M GuHCl, 20 mM EDTA, 50 mM Tris-HCl pH 7.0, and 1% diatomaceous earth were added to FS, S1 and S2 fractions. After incubating for 5 min at room temperature, the mixture was centrifuged as described above. The supernatant was discarded and the pellet was washed four times with wash buffer (50 mM Tris-HCl pH 7.4, 200 mM NaCl, 10 mM EDTA and 50% ethanol). The pellet was rinsed with acetone and dried briefly at 65°C. DNA was eluted by resuspending diatoms in water (10% of original supernatant volume) and heating at 65°C for 10 min.

### PCR

Sample processing and PCR were carried out in dedicated areas using dedicated pipettes and filter guard tips to prevent cross contamination. *M. tuberculosis *DNA was detected by PCR using primers targeting IS*6110 *[14]. Briefly a 40-μl reaction was set up containing 0.5 μM each of primers T4 (5' CCTGCGAGCGTAGG CGTCGG 3') and T5 (5' CTCGTCCAGCGCCGCTTCGG 3'), 1.5 mM MgCl_2_, 0.2 mM dNTPs, 1 U of Taq DNA polymerase (GeneTaq, MBI Fermentas, Vilnius, Lithuania) and 10 μl of template DNA in case of sediment fractions or 4 μl of template DNA in case of supernatant fractions. In case of supernatants, neat, 1:5 and 1:10 dilutions were subjected to amplification. DNA negative and positive control reactions without DNA and containing 10 to 20 ng of *M. tuberculosis *H37Rv DNA, respectively were routinely set up. The thermal cycling parameters were initial denaturation at 94°C for 10 minutes followed by 45 cycles each of 1 minute at 94°C and 1 minute and 30 seconds at 60°C with a final extension of 5 minutes at 72°C. The 123-bp PCR product was detected by electrophoresis on 2.3% agarose gel followed by ethidium bromide staining and visualization under UV light. For the experiment described in Fig. 2, only 35 cycles of amplification were used. The *devR *PCR assay yielded a 164-bp amplification product [10].

### Statistical analysis

The significance of the decrease in smear grade status and of the increase in supernatant PCR positivity was determined by McNemar's chi square test using Stata 9.0 software (StataCorp LP, College Station, Texas, USA). The significance of the decrease in bacterial load observed during storage was determined using the nonparametric Friedman Anova test followed by multiple comparison. P values of < 0.05 were considered significant (SPSS 10.0.1 software, Chicago, USA). The median, interquartile values and box plots (Fig. 6) were calculated and generated using Stata 9.0 software.

## Authors' contributions

MH provided the smear positive sputum samples (and their direct smear status) collected from patients before they were placed on antitubercular therapy. DP and SC carried out sample processing, PCR and smear microscopy, participated in designing the study, partial analysis of the data and drafting the manuscript. JST conceived of the study, and participated in its design and coordination, performed the statistical analysis and finalized the manuscript. All authors read and approved the final manuscript.
